# Comparison of LAL and rFC Assays—Participation in a Proficiency Test Program between 2014 and 2019

**DOI:** 10.3390/microorganisms8030418

**Published:** 2020-03-16

**Authors:** Maike Piehler, Ruth Roeder, Sina Blessing, Johannes Reich

**Affiliations:** Endotoxin Test Service, Microcoat Biotechnologie GmbH, Am Neuland 3, 82347 Bernried am Starnberger See, Germany; m.piehler@microcoat.de (M.P.); r.roeder@microcoat.de (R.R.); s.blessing@microcoat.de (S.B.)

**Keywords:** bacterial endotoxin testing (BET), endotoxin, lipopolysaccharide (LPS), *Limulus* Amebocyte Lysate (LAL), recombinant Factor C (rFC), proficiency testing

## Abstract

Endotoxin (lipopolysaccharide) testing of drugs is routinely required in pharmaceutical industries. Suitable compendial assays are defined by national pharmacopoeias. At this time, *Limulus* Amoebocyte Lysate (LAL) assays are the gold standard. LAL is used in vitro for specific detection of endotoxin based on endotoxin-activated Factor C-mediated clotting cascade. However, alternative mediated pathways (e.g., Factor G), impurities, and further factors may influence test results. Some of these influencing factors are eliminated by recombinant Factor C (rFC) test, which represents a promising alternative. rFC not only enables highly specific endotoxin testing, as interfering Horseshoe Crab blood components are eliminated, but also offers ethical and ecological advantages compared to classical LAL assays. However, the question remains whether rFC-based tests are robust test systems, equivalent or superior to LAL and suitable for routine bacterial endotoxin testing. Pharmaceutical test users have validated the test successfully for their specific products, but no long-term studies have been published that combine testing of unknown samples, inter-laboratory, -operator, and -lot changes. Thus, it was of great interest to investigate rFC test performance in a routine setting within a proficiency test program set-up. Over a period of six years comparative endotoxin testing was conducted with one kinetic chromogenic LAL assay and two rFC-based assays. Results of this study demonstrate that both rFC-based assays were comparable to LAL. All results met acceptance criteria defined by compendial bacterial endotoxin testing. RFC-based methods generated results with even better endotoxin recovery rates compared to LAL. Therefore, rFC-based tests were found to represent reliable methods, as equivalent or even superior to LAL assays and suitable for routine bacterial endotoxin testing.

## 1. Introduction

**Figure 1:**
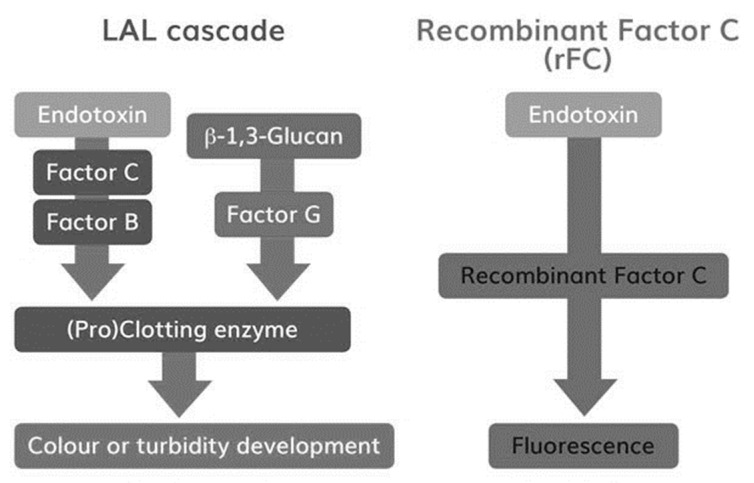
Reaction cascades in *Limulus* Amoebocyte Lysate (LAL) and recombinant Factor C (rFC) assays.

Drugs for parenteral administration require strict testing for contamination with regards to bacterial endotoxin. These can induce life-threatening inflammatory reactions after injection or infusion into the blood stream or interfere with the drug, leading to undesired side effects. In earlier days, samples were routinely injected into rabbits to examine the potential for endotoxin contamination. Over 30 years ago the Rabbit Pyrogen Test (RPT) was mostly replaced by *Limulus* Amoebocyte Lysate (LAL) assays for detection of endotoxin. LAL, using lysate of blood cells (i.e., amoebocytes) from Horseshoe Crabs, is sensitive for bacterial endotoxins [1,2,3]. Endotoxin specifically activates zymogen Factor C, the protein responsible for blood coagulation in Horseshoe Crabs [4]. Thus, instead of injecting samples into rabbits, blood is drawn and purified from Horseshoe Crabs for in vitro endotoxin-specific coagulation testing. Today, various test setups, from pure manual coagulation tests (i.e., gel-clot) to computerized kinetic chromogenic tests, are available. However, independent of the test setup the full coagulation cascade is represented in such assays including an alternative Factor G-mediated pathway (Figure 1). Via this pathway beta-glucans can activate LAL, leading to test interference [5,6]. Sample impurities and inadequate sample conditions like pH and temperature can further decrease specificity by influencing the reaction cascade [4]. In contrast, recombinant Factor C (rFC) assays are purely endotoxin-specific, as all additional Horseshoe Crab blood constituents are eliminated. Activated Factor C reacts directly with a fluorophore (Figure 1) [7]. Fluorescence detection delivers highly sensitive and accurate results. Even though rFC assays have been commercially available since 2003, LAL remains the compendial method when it comes to endotoxin testing for drug release. Although rFC assays are already used in various cases [8,9,10,11,12,13], rFC still lacks worldwide acceptance as a compendial test method for release testing. Its reaction mechanism with endotoxin is equivalent to the classical LAL test, thus rFC represents a promising chance to improve and modernize bacterial endotoxin testing (BET) for pharmaceutical quality control. The recombinant Factor C is biotechnologically engineered and thus a protein of high purity and low inter-lot variability. rFC enables assays with high endotoxin-specificity [11,12]. Hence, rFC-based tests have the potential to increase sensitivity and accuracy of classical bacterial endotoxin detection. The recombinant protein can easily and sustainably be produced in unlimited amounts without the use of animals, thus conserving vulnerable populations of Horseshoe Crabs. As the endotoxin testing is ubiquitously required for drug safety testing by pharmaceutical companies, the full acceptance of rFC as a compendial method would considerably contribute to the effort to eliminate animal-based tests in support of the European Directive 2010/63/EU for the protection of animals used for scientific purposes and consistent with the 3 Rs principle to reduce, refine or replace animals in testing. 

Figure 1: Reaction cascades in LAL and rFC assays. The figure depicts the high-level difference in reaction pathways of LAL and rFC. Besides the endotoxin-specific pathway, a beta-Glucan reaction pathway is given in the LAL cascade. Simplification of the classical LAL test milieu provides a first level of specificity for endotoxin testing via rFC-based methods [14].

To this end, it should be clarified whether rFC represents a robust equivalent to LAL or an even superior method for routine quality control endotoxin testing in pharmaceutical industries. Bolden et al. [12] showed that the use of rFC is a robust replacement of LAL for BET and can be validated for the detection of bacterial endotoxins in a variety of pharmaceutical products. However, so far no long-term studies have been published that combine the testing of samples of unknown composition and contaminations using rFC assays. Within such a study various lots of the individual reagents are obtained. Further, tests are performed by different operators in different labs. Thus, it was of significant interest to investigate and summarize the results obtained from the participation of an LAL routine proficiency test program in comparison with rFC-based methods performed over six years (2014 to 2019) at different laboratories at Microcoat. The outcome will help to assess whether rFC assays detect endotoxin in such unknown samples with similar specificity, accuracy and precision as compendial LAL over several years, across different in-house laboratories, and with steady inter-operator and inter-lot stability. 

## 2. Methods

For detection of endotoxin, three different bacterial endotoxin test methods were used, one LAL and two rFC-based assays. 

### 2.1. LAL–Kinetic Chromogenic LAL Assay

The kinetic chromogenic LAL assay (Endosafe Endochrome-K™, Charles River Laboratories) was used according to the manufacturer’s instructions. In the following manuscript, LAL is referred to as Endosafe Endochrome-K™. The absorption at 405 nm was measured using an Elx808 reader (BioTek Instruments GmbH, Bad Friedrichshall, Germany). All samples were measured in duplicate and average values were used. Standard curves were fitted using a linear regression model. The detection limit of the assay was 0.005 EU/mL. In order to control test interference, positive product controls (PPC) according to manufacturers’ instructions were performed. Data of each run were regarded as valid data only if the following acceptance criteria were met:

- The temperature during the measurement must be 37 ± 1.0 °C.
- Fit of the standard curve: r ≤ −0.980.
- Linear regression: Slope must be between −0.400 and −0.100.
- Linear regression: Y Intercept has to be between 2.500 and 3.500.
- The mean onset-time of the blank must be higher than the mean onset-time of the lowest standard concentration.
- The Coefficient of Variation (CV) of all replicates must be ≤10%.
- The PPC must be between 50% and 200%. Therefore, 0.5 EU/mL Control Standard Endotoxin (CSE) was spiked into the diluted and undiluted sample, respectively.

### 2.2. rFC-Based Assays

As alternative to the LAL test, two tests based on recombinant Factor C (ENDOZYME^®^, ENDOLISA^®^, Hyglos GmbH, a bioMérieux company, Bernried, Germany) were used. Both were used according to manufacturer’s instructions. ENDOZYME^®^ is a homogenous rFC-based test and is prepared like a classical LAL test. In the following manuscript the rFC test is referred to as ENDOZYME^®^. Other than LAL and rFC tests, ENDOLISA^®^ is based on a heterogenous test format and deviates in preparation. ENDOLISA^®^ includes an additional endotoxin binding step based on ligands from bacteriophages, as well as washing steps before the actual detection step using rFC [15]. This test procedure is similar to ELISA test preparations. In the following manuscript Endolisa is referred to ENDOLISA^®^.

The released amount of fluorescence substrate in both assays was measured fluorometrically at 440 nm (Excitation: 380 nm) with a FLx800 fluorescence microplate reader (BioTek Instruments GmbH, Bad Friedrichshall, Germany). All samples were measured in duplicate and average values were used for further calculations. 

### 2.3. rFC Test

Standard curves were fitted using a 4-parameter logistic nonlinear regression model. Since 2018, a linear regression model has been used. The detection limit of the assay was 0.005 EU/mL. In order to control test interference, positive product controls according manufacturer’s instructions were performed. Data of each run were regarded as valid data only if the following acceptance criteria were met:

- The temperature during the measurement must be 37 ± 1 °C.
- Fit of the standard curve: r > 0.980.
- Quality of the standard curve: Back Calculated Concentrations (BCCs) 75% to 133% for standards 5 to 0.005 EU/mL.
- The blank must be smaller than the lowest standard.
- The CV of all replicates must be ≤25%.
- The PPC must be between 50% and 200%. Therefore, 0.5 EU/mL Control Standard Endotoxin (CSE) was spiked into the diluted and undiluted sample, respectively.

### 2.4. Endolisa

Standard curves were fitted using a 4-parameter logistic nonlinear regression model. The detection limit of the assay was 0.05 EU/mL. In order to control test interference, positive product controls according manufacturer’s instructions were performed. Data of each run were regarded as valid data only if the following acceptance criteria were met:

- The temperature during the measurement has to be 37 ± 1 °C.
- Fit of the standard curve: r > 0.980.
- Quality of the standard curve: BCCs 75% to 133% for standards 50 to 0.05 EU/mL.
- The blank must be smaller than the lowest standard.
- The CV of all replicates must be ≤25%.
- The PPC must be between 50% and 200%. Therefore, 5.0 EU/mL Control Standard Endotoxin (CSE) was spiked into the diluted and undiluted sample, respectively.

### 2.5. Sample Preparation and Calculation of Recovery

All tested samples were of unknown composition and obtained as part of the LAL Proficiency Testing Program from Charles River Laboratories. The samples were received as lyophilized material and reconstituted according to the sponsor’s requirement in water dedicated to endotoxin testing (Hyglos GmbH, a bioMérieux company, Bernried, Germany) and mixed by vortexing for at least 5 min. Bacterial endotoxin testing using LAL and rFC-based test systems was performed as described above. The samples were measured undiluted or in at least two dilutions in order to overcome potential test interference. For interpretation of measured results, first valid dilutions were used. Obtained results of each assay were rated as valid when PPC recovery was between 50% and 200%.

Sample recovery:

Sample recovery (%) = (Determined Value (EU/mL))/(Nominal Value (EU/mL)) × 100(1)

Back calculation of standards:

Transformation of standards, measured from onset times [seconds] and relative fluorescence units [RFUs] to endotoxin units (EUs/mL) using respective standard curves resulting in determined standard value.

Normalized back calculated Standard = (Determined Standard Value (EU/mL))/(Nominal Standard Value) × 100(2)

### 2.6. Software

Standard curves and endotoxin concentrations were calculated with Gen5 Data Analysis Software Version 2.05 from BioTek Instruments GmbH, Bad Friedrichshall, Germany. For calculation of endotoxin recovery and plots Microsoft Office Home and Buisness 2016 was used.

## 3. Results

As part of a proficiency test program over a period of six years, from March 2014 until June 2019, a conventional LAL assay was compared with an rFC test and Endolisa. During this time period, 13 samples with unknown composition and endotoxin concentrations were received. Thirteen samples were analyzed with LAL, 11 samples were analyzed with the rFC test and 11 samples were analyzed with Endolisa. All of the analyses were performed either with LAL or rFC-based assays and led to valid results. As this summary was not planned beforehand, some samples were not analyzed with all methods. 

**Figure 2:**
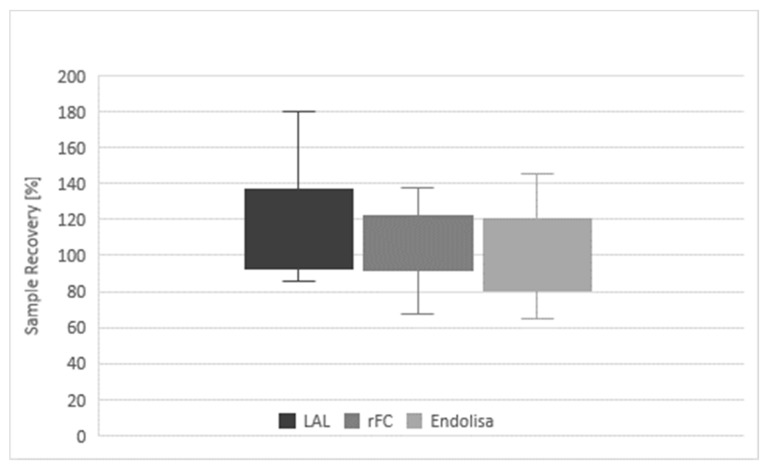
Comparison of sample recovery and PPC.

In Table 1 the determined values are given for all of the analyses. In addition, the sample recovery in relation to the respective nominal concentration is calculated, which was provided by the sponsor after results were submitted. With LAL, 13 samples were analyzed and resulted in a mean recovery of 120%, compared to the provided nominal values (100%). With the rFC test and Endolisa, 11 samples were analyzed and the mean recovery was 107% and 100%, respectively. The corresponding mean PPCs within the assays were 113% for LAL, 105% for the rFC test and 96% for Endolisa (Table 2). The corresponding coefficients of variation were 23%, 18% and 23% for LAL, rFC test and Endolisa, respectively. The overall mean sample recovery was 110%, slightly above the expected 100%. A further summary of these results is given in Figure 2, reflecting the measured minimum and maximum values as well as the lower and upper quartile of each method. 

Table 1: Results of LAL and two rFC-based assays. The table shows test results obtained from 13 proficiency testings between 2014 and 2019. For bacterial endotoxin detection LAL and two rFC-based detection methods were used. The sample composition as well as the contamination were unknown. The nominal values were provided by the sponsor of the program after submission of the LAL test result. Positive Product Controls (PPC) were used to verify validity of the analysis. For calculation of sample recovery, the determined endotoxin values were referred to the nominal value and stated in percent (Equation (1)). Not all samples were analyzed with all of the three methods. Assays not performed are indicated n.t. (not tested)).

Figure 2: Diagram of results of LAL and recombinant assays. Overall sample recovery, differentiated by the individual test methods (LAL, rFC and EndoLISA) is given. The boxplots represent the lower (Q1) and upper (Q3) quartile and the minimum and maximum range. 

**Figure 3:**
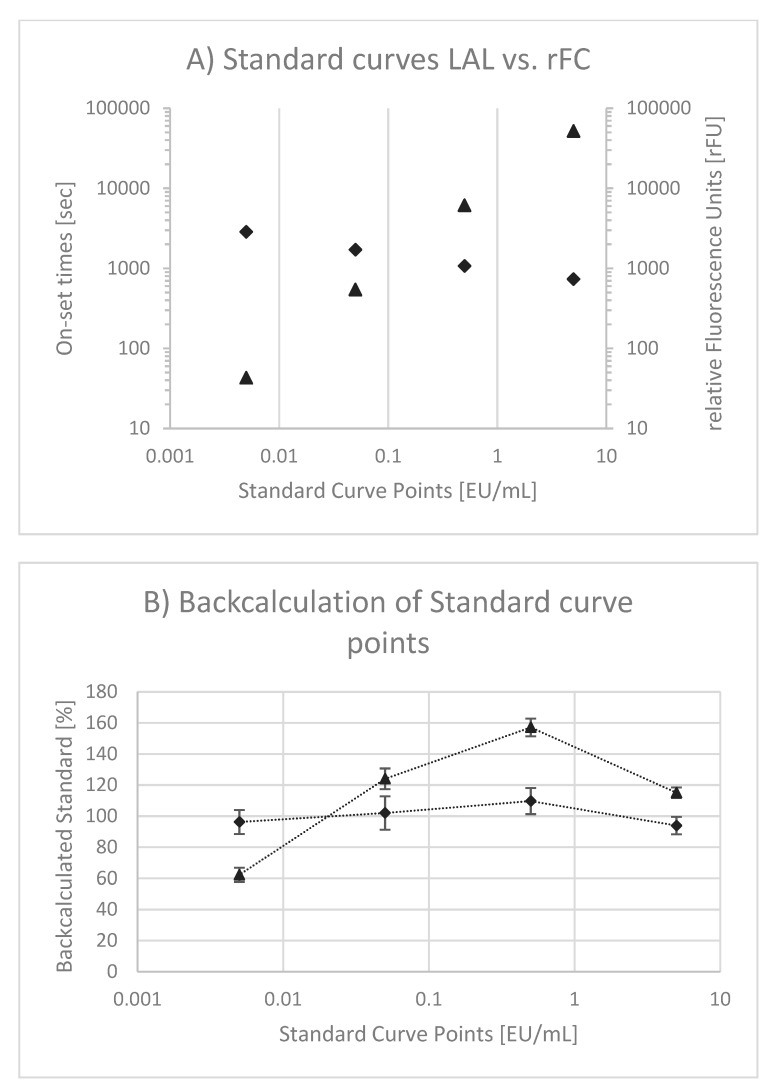
Linearity of Standard Curves. In (**A**), typical endotoxin standard curves using a kinetic chromogenic LAL test (triangle) and an rFC test (diamonds) are given. For LAL onset times in seconds and for the rFC test relative fluorescence units are plotted as functions of endotoxin concentrations in EU/mL, respectively. In (**B**) back-calculated measured standard curve points are plotted as a function of the nominal standard curve point and are normalized to 100. The data points for LAL (triangle) and rFC (diamonds) are mean values out of individual standard curve measurements (*n* = 9), each. The error bars reflect the standard deviation of replicates.

Although the specific interactions between endotoxin and Factor C of LAL and rFC-based assays are similar, differences are given in read-out (i.e., optical density vs. fluorescence) and slope of standard curves (i.e., negative vs. positive). Obviously, standard curves using LAL and rFC are different (Figure 3A). In both cases double logarithmic scales are used. In case of LAL, onset times in seconds are plotted as a function of the endotoxin concentration. As onset times decrease (e.g., 2860, 1711, 1070 and 734 s) when endotoxin concentrations are increased (0.005, 0.05, 0.5 and 5 EU/mL), a negative slope (−0.198) is observed. Although the onset times range only from 734 to 2860 s, a logarithmic scale is used according to standardized LAL test procedures. In the case of rFC, relative fluorescence units (rFUs) are plotted as a function of the endotoxin concentration. The rFUs (e.g., 51, 552, 6134 and 51,968 rFU) increase when endotoxin concentrations (0.005, 0.05, 0.5 and 5 EU/mL) are increased. Consequently, a positive slope (+1.006) results. For both test methods, a linear correlation of signal (onset and rFU, respectively) and endotoxin concentration is observed. However, the linear correlation in LAL has been questioned [16]. In order to prove this observation and to compare whether the same behavior is given within the rFC test, measured standard curve points from 0.005 to 5 EU/mL were back-calculated to EU/mL and normalized to 100% (Equation (2)). The result is presented in Figure 3B. The back-calculated standard curve points of LAL confirm the previous described curve behavior because of a hyperbole curve shape (the so-called “bow in the curve”). In contrast, for rFC test variations around 100% without a hyperbole curve shape are observed. 

## 4. Discussion

The resulting data from proficiency testing over six years provides a unique data review in comparing classical LAL and next generation rFC-based test methods. Overall, all nominal sample concentrations could validly be determined by the LAL assay, rFC test and Endolisa. In all cases, the particular measurements showed reliable detection of endotoxin independent of the used method. No failure was observed. The mean recovery rates of 107% (rFC test) and 100% (Endolisa) are closer to 100% of the nominal value than the mean recovery rate of 120% obtained for LAL. Both rFC-based assays obtained lower minimum recovery rates, but also lower maximum recovery rates that were closer to 100% than LAL. A similar result is obtained comparing the mean PPCs of the three different assays (Table 2). The bandwidth of recovery is reflected by the coefficient of variation, which tends to smaller variations in rFC-based assays compared to LAL assays. The data indicate that both rFC-based assays deviated less from the nominal value than LAL and showed smaller or similar CVs (Figure 2). Thus, accuracy and precision of both rFC-based assays were equivalent or higher than was observed for the LAL endotoxin assay. 

Table 2: Summary of proficiency test program results. The table shows mean recoveries and coefficients of variation (CVs) of the samples and the PPCs, with respect to the individual test methods. In total, 13 samples were analyzed using 35 analysis. Thirteen samples were analyzed with LAL, and 11 samples were analyzed with the rFC test and Endolisa. 

A higher accuracy and precision of rFC-based methods might be explained by the reaction cascade and standard curve interpretation. In case of LAL assays (see Figure 1), endotoxin activates Factor C, which in turn triggers the full reaction cascade (i.e., Factor B, Proclotting). Finally, the turnover of a chromogenic substrate is measured by optical density (OD). The resulting reaction kinetics of the individual standards (OD as function of time) are very fast. In order to obtain a broad dynamic test range, an OD threshold is defined for kinetic analysis, because an end-point analysis would result in a small dynamic test range. This threshold is used to measure the period of time until the threshold is reached. It is called onset time. To calculate endotoxin concentrations, standard curves are plotted using onset times as a function of the endotoxin concentration in a double logarithmic manner. This relation is intended to be linear. Due to the double logarithmic scale, deviations from linear behavior are difficult to perceive. It was described that hyperbole standard curve shapes are likely [16] and our results given in Figure 3B confirm this behavior. In consequence, the calculation of endotoxin might be over- or underestimated by using a linear model. This nonlinear curve behavior might be explained by the complex reaction cascade of LAL and one reason for the deviation from nominal values using a linear fitting model, as most commonly used. To this end, a nonlinear fit model that describes observed standard curve behavior might be recommended.

In the case of the rFC test, endotoxin activates Factor C, which in turn directly triggers the turnover of a fluorogenic substrate. Other than OD measurements with a dynamic range from 0.2 to 4 units (~2 log), fluorescence measurements allow a broad range of detection from approximately 10 to 100,000 units (4 log). Due to this fact, a broad dynamic test range is obtained by analysis of the endpoints. Using onset time is an elegant method of extending the dynamic range, but this is not needed in the case of rFC-based assays. 

To compare standard curves between the rFC test and LAL, the rFC test does not show hyperbole curve behavior. This might explain the higher precision of rFC-based assays compared to LAL. It can be further observed that the slope in rFC tests was approximately +1.0 whereas the slope in LAL was approximately –0.2. The latter is a result of a complex reaction cascade plus transformation into onset times. The slope of 1.0 in rFC tests reflects a more defined reaction between endotoxin and Factor C, because an increased endotoxin concentration leads to the same increase in detectable substrate turnover. For example, a 10-times higher endotoxin concentration leads to a 10-times higher rFU. This has the benefit that variations in rFU are in the same range as variations in EU/mL. In contrast, a small slope in the LAL standard curve magnifies variations that are obtained in onset times and transformed into concentrations (i.e., EU/mL). For example, a slope of 0.2 magnifies a given variation by factor 5. 

Apparently, comparison of two rFC-based assays and LAL show very comparable results. Considering the test procedures of the individual assays, the LAL and the rFC test are equivalent in sample preparation and test procedure. Both are based on a homogeneous test format, meaning the sample is directly mixed with the reagent (i.e., lysate or rFC reagent, respectively). Furthermore, in both cases, Factor C, native or recombinant, reacts directly with endotoxin. Similar test results were expected and are confirmed. However, the Endolisa method follows a heterogeneous test format. In a first step, endotoxin is bound to a solid phase (i.e., phage ligands) and supernatants are washed away in the second step. In the final step, only bound endotoxin is detected via rFC [15]. Different from a homogeneous test format, specificity and sensitivity of the assay are pre-determined by the phage ligand in a heterogeneous test format. Thus, there is a possibility that homogeneous LAL/rFC and heterogeneous Endolisa show different results because of the origin of the respective molecules (Horseshoe Crab vs. Bacteriophage). Varying test results between Endolisa and LAL have been discussed [17], but within the present study, no substantial discrepancies between the methods are observed. It is important to note that all of the test systems represent models. For example, Perdomo-Morales et al. demonstrated varying test results by comparing LAL, Rabbit Pyrogen Test and the Monocyte Activation Test [18]. Apparently, models derived from Horseshoe Crabs, Rabbits, Bacteriophages and Humans are different, which can lead to deviating results detecting endotoxin. 

Taken together, LAL and the rFC test, as well Endolisa, obtained valid results for all tested samples based on the common acceptance criteria of 50% to 200%, as generally specified by pharmacopoeias [19,20,21]. Comparability of all tested assays is given. Considering accuracy and precision, the rFC test and Endolisa obtained results with recovery rates closer to 100%, with equal or smaller CVs than LAL. This shows that rFC represents a very reliable model, equivalent or even superior to LAL and suitable for routine bacterial endotoxin testing. 

## Figures and Tables

**Table 1:** Result of six years proficiency test program participation.

PTP Study (Quarter, Year)	Nominal Value (EU/mL)	Method	Value Determined (EU/mL)	Positive Product Control (PPC) (%)	Sample Recovery (%)
Q1, 2014	0.2135	LAL	0.385	117	180
		rFC	0.294	129	138
		Endolisa	0.256	95	120
Q2, 2014	1.376	LAL	1.549	108	113
		rFC	1.605	75	117
		Endolisa	1.995	85	145
Q3, 2014	0.603	LAL	0.634	97	105
		rFC	0.735	101	122
		Endolisa	0.485	94	80
Q4, 2014	0.2784	LAL	0.440	136	158
		rFC	0.270	93	97
		Endolisa	0.335	76	120
Q2, 2015	1.195	LAL	1.082	79	91
		rFC	0.808	97	68
		Endolisa	1.060	91	89
Q4, 2015	0.19	LAL	0.255	79	134
		rFC	0.157	118	83
		Endolisa	0.123	77	65
Q2, 2016	16.79	LAL	15.372	92	92
		rFC	19.386	153	115
		Endolisa	15.449	97	92
Q4, 2016	21.84	LAL	30.568	127	140
		rFC	23.892	104	109
		Endolisa	15.622	105	72
Q2, 2017	1.333	LAL	1.496	100	112
		rFC	n.t.	n.t.	n.t.
		Endolisa	1.290	126	97
Q4, 2017	1.375	LAL	1.822	119	133
		rFC	n.t.	n.t.	n.t.
		Endolisa	n.t.	n.t.	n.t.
Q2, 2018	25.999	LAL	24.300	108	93
		rFC	29.780	103	115
		Endolisa	n.t.	n.t.	n.t.
Q4, 2018	18.608	LAL	16.000	179	86
		rFC	23.450	95	126
		Endolisa	20.192	104	109
Q2, 2019	16.172	LAL	19.260	126	119
		rFC	14.780	83	91
		Endolisa	17.310	110	107

**Table 2:** Summary of results.

Assays	PPC (%)	Sample Recovery (%)
Mean (all) *n* = 35	105.1	109.5
CV (all)	21.0	22.9
Mean (LAL) *n* = 13	112.8	119.7
CV (LAL)	22.7	22.8
Mean (rFC) *n* = 11	104.6	107.3
CV (rFC)	19.9	18.3
Mean (EndoLISA) *n* = 11	96.4	99.5
CV (Endolisa)	14.5	22.7
